# Effects of the discharge of uranium mining effluents on the water quality of the reservoir: an integrative chemical and ecotoxicological assessment

**DOI:** 10.1038/s41598-017-14100-w

**Published:** 2017-10-24

**Authors:** Carla Rolim Ferrari, Heliana de Azevedo Franco do Nascimento, Suzelei Rodgher, Tito Almeida, Armando Luiz Bruschi, Marcos Roberto Lopes do Nascimento, Rodrigo Leandro Bonifácio

**Affiliations:** 1 Radioecology Laboratory, Poços de Caldas Laboratory, ‖ Brazilian Nuclear Energy Commission, Rodovia Poços de Caldas/Andradas km 13, Poços de Caldas, MG 37719-005 Brazil; 2 São Paulo State University (UNESP). Institute of Science and Technology, São José dos Campos. Rodovia Presidente Dutra, Km 137, 8 Eugenio de Melo, São José dos Campos SP, 12247-004 Brazil; 3 Community Ecology Laboratory, University of Vale do Itajaí, Rua Uruguay, 458, Itajaí, SC 88302-202 Brazil; 4 Chemical Analyses Laboratory, Poços de Caldas Laboratory, Brazilian Nuclear Energy Commission, Rodovia Poços de Caldas/Andradas km 13, Poços de Caldas, MG 37719-005 Brazil

## Abstract

The water quality of the Antas reservoir, under the influence of treated effluents from a uranium mining area Ore Treatment Unit (UTM) with acid mine drainage, was investigated. Samples were collected every 3 months from the Antas reservoir (CAB, P41-E and P14) and from the UTM (P41-S). Chemical and acute 48 h toxicity tests using *Ceriodaphnia silvestrii* and *Daphnia magna* analyses were carried out to determine the potential environmental risks due to discharging the uranium mine effluents into this reservoir. All the water samples taken from the treated effluent (P41-S) were positively correlated with elevated concentrations of uranium, manganese, aluminum, zinc and fluoride and with high electrical conductivity and pH values, being considered toxic. In November 2014 water samples taken from the reservoir showed chemical concentrations above the legislation limits for fluoride (4.5 mg L^−1^) uranium (0.082 mg L^−1^), sulfate (662.4 mg L^−1^), manganese (1.125 mg L^−1^) and aluminum (1.55 mg L^−1^), and in July 2015 for fluoride (2.55 mg L^−1^), uranium (0.01 mg L^−1^) and manganese (0.36 mg L^−1^). The extremely high average value for hardness (543.55 mg L^−1^) possibly reduced the toxicity potential of this chemical species mixture with respect to the bioindicators. The influence of the variation in water hardness on the toxicity of the cladocerans was discussed.

## Introduction

Historically mine sites are a major source of contamination to aquatic environments and countries throughout the world face severe environmental problems due to deactivated uranium mines. Environmental surveys carried out around U mining sites are generally based on physical, chemical and dosimetric measurements, without taking biological effects into account^1,2^. The simultaneous presence of a large number of radioactive and stable chemical species in uranium mine effluents increases the challenge of assessing the toxicity of such complex mixtures, whose effects on the environment are still not fully understood^3^. Ecotoxicological research and the effects of these chemicals on the aquatic ecosystems surrounding mining areas have mainly focused on temperate countries, while little information is available about tropical ecosystems. The few studies carried out in aquatic systems situated in uranium mining regions, including ecotoxicological analyses and physical and chemical parameters, were carried out in Australia^4^, Portugal^3^, Canada^5,6^, the United States of America^7^ and the Czech Republic^8^, indicating the need to expand the knowledge concerning the ecotoxicological approach to these particular situations outside these countries.

In Brazil (Caldas, Minas Gerais State) there is a uranium ore-mining area (Ore Treatment Unit of the Brazilian Nuclear Industries - UTM/INB), the main environmental problem of which is the generation of acid mine drainage (AMD) containing high concentrations of fluoride, sulfate, manganese, zinc, aluminum and uranium. Before being discharged into the environment (Antas reservoir), this uranium mine effluent is treated with slaked lime, contributing to the high hardness values registered in the water samples taken from the Antas reservoir^9,10^. AMD is frequently observed in mine sites that contain sulfide rocks, being caused by the oxidation of metal sulfides (mainly pyrite) to produce sulfuric acid and discharge metals, with potential toxicity^11^. This represents an important source of water quality degradation throughout the world^12^, since its continuous release into the environment is related to a severe pollution problem associated with these mining activities. Thus much attention has been paid to the degradation of aquatic ecosystems downstream from mine sites affected by AMD^12–15^.

The aquatic environment usually represents the final destination of contaminants from problematic areas, where they can affect the local biota^16^. In studies about the assessment of environmental contamination, the integrated approach between chemical analyses and toxicity tests is considered to be an efficient strategy to better comprehend the ecological effects of releasing treated effluents into the freshwater system^17,18^. Recently, authors have suggested that an ecotoxicological characterization of the aquatic environment under the influence of the UTM/INB should be carried out, in order to better assess the risk of toxic effects and consequences of the chemical species in this particular case, with respect to the aquatic biota^9,10^. According to Goulet *et al*.^19^, more toxicity studies with water chemistry downstream from U mines and mills are necessary, to support predictive assessment of the impacts of U discharge into the environment.

In this context, the present study was designed to evaluate the relation between the chemical and ecotoxicological approaches, taking seasonal and spatial samples from the reservoir under the influence of treated effluents coming from the uranium mine and containing AMD. Acute 48 h toxicity tests were carried out with *Ceriodaphnia silvestrii* and *Daphnia magna*, to assess possible risks caused by the uranium mine effluents discharged into the tropical freshwater systems.

## Materials and Methods

### Study sites

The UTM/INB was the first uranium deposit to be exploited in Brazil and is located on the Poços de Caldas Plateau (Minas Gerais State, Brazil) at 1291 m above sea level. Uranium production in this mining area started in 1982 and lasted for 13 years, generating a total of 1242 tons of U_3_O_8_^20^. During the development of the mine, 44.8 10^6^ m^3^ of rock were removed, and of this amount, 10 M tons were used as building material (roads, ponds, etc.), the rest being disposed of in two major waste rock piles. Mining activities no longer take place at this site, but the chemical plant in charge of the liquid effluent treatment is still active^21^. The acid effluent is treated by chemical processes with calcium hydroxide and oxide (slaked lime) and flocculating agents, and then directed into a decantation basin trough for sedimentation of the stable and radioactive metals. After treatment (P41-S site), the effluent is then discharged into the Antas reservoir (in a same rater in the rainy and dry season of the 0.08 m^3^ s^−1^, totalizing 2.6 10^6^ m^3^ y^−1^), which presents a unidirectional water flow. The Antas reservoir is located in the southeast of Minas Gerais State and has a drainage area of 51 Km^2^, a maximum length of 3500 m, a volume of 3.9 × 10^6^ m^3^, a flow rate of 9.52 m^3^ s^−1^ and an average depth of 4 m (maximum of 8 m)^22^. According to the trophic state index, the Antas reservoir has been classified as an oligotrophic environment^9^.

Climatically, the region is classified as Cwb according to the Köppen criteria, between group A (tropical and hot) and group C (mesothermic conditions - dry winter and rainy summer). The minimum and maximum temperatures vary between 12 °C and 25 °C and the average annual rainfall is around 1,700 mm^23^. In this region, the rainy season lasts from October to March and the dry season from April to August^22^.

**Figure 1:**
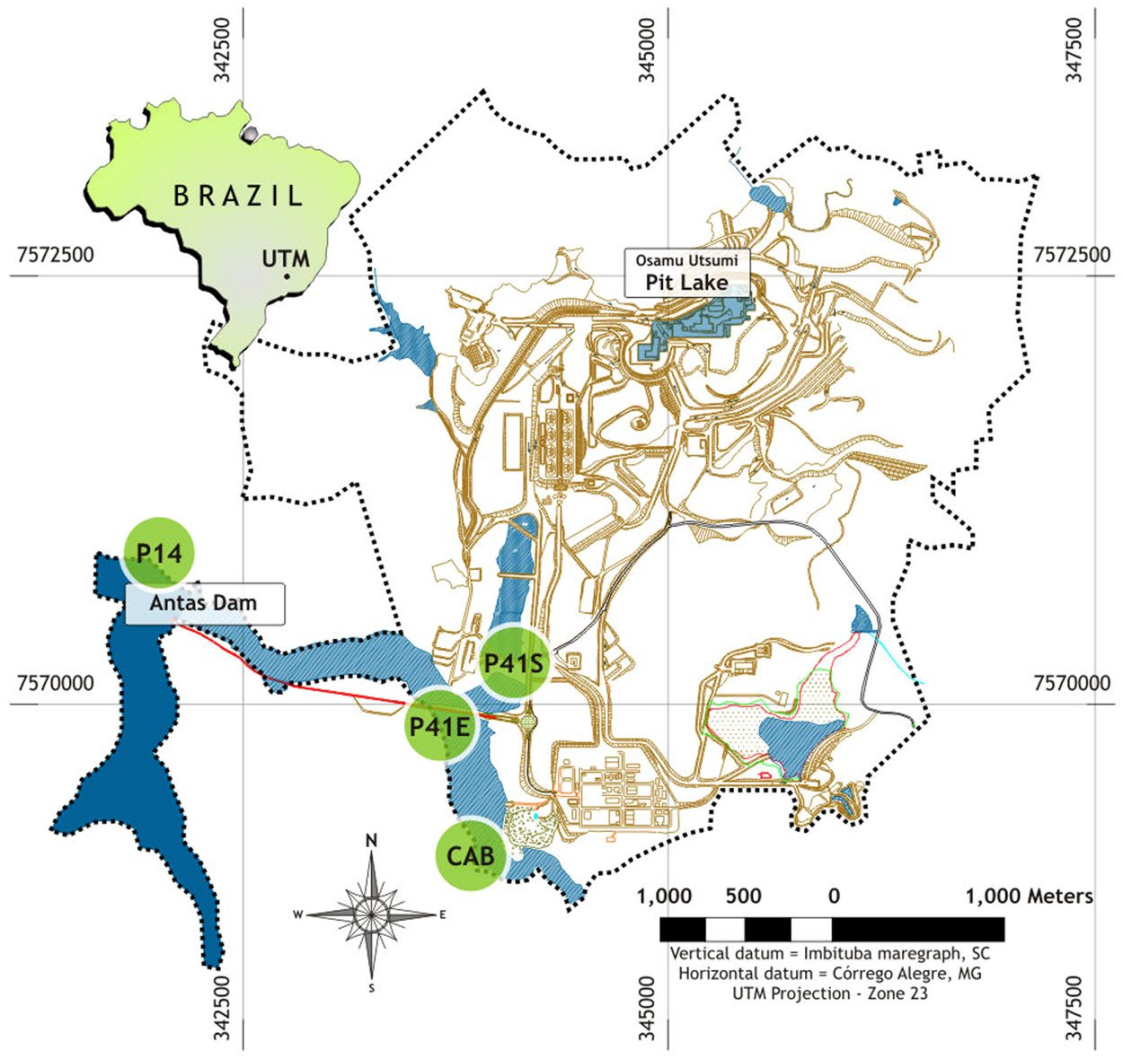
Locations of the sampling sites in the Ore Treatment Unit/Brazilian Nuclear Industries (UTM/INB) and in the Antas reservoir. Figure 1 was produced in ArcGIS software version 9.3.1, by Alberti H.L.C. and Filho E.O.L.).

The present study involved the evaluation of three sampling sites in the pelagic zone within the Antas reservoir (CAB, P41-E and P14), and one site in an artificial pond in the UTM/INB area (P41-S). The P41-S site corresponded to treated effluent, before being discharged into the Antas reservoir. The reference site, CAB site, 1.5 m deep, was located at the head of the reservoir upstream and about 1500 m from the mining effluent discharge zone; The P41-E site was 1.0 m shallower and located near the effluent discharge zone, and P14 was downstream of P41-E near the reservoir dam, and deeper than the other sites, about 6.9 m deep and located about 2000 m from of the effluent discharge zone^9^ (Fig. 1). In the Antas reservoir, the unidirectional water flow is from the CAB site to the P14 site. The proposed water sample collection locations are sites from nonpoint sources, which were previously defined and discussed with the Authorization Officer and the Operator. For each sampling site there are Brazilian guidelines related to water quality^24–26^.

Sampling was carried out during four periods: November 2014, February, July and October 2015. The water samples were collected using 5-L Van Dorn bottles. The physical and chemical analyses and acute 48 h toxicity tests were carried out on all the water samples from the Antas reservoir (CAB, P41-E and P14 sites) and on the treated effluent (P41-S site).

### Physical and Chemical Analyses

The electrical conductivity, dissolved oxygen, pH and temperature of the water were measured using a Horiba^®^, model U-10 multiparameter probe field meter. The reduction-oxidation potential was determined using a Digimed^®^ model DM-22 Pt electrode (with Ag/AgCl as the reference). Water samples were taken to determine the chlorophyll *a* concentration according to Lorenzen^27^. The total suspended solids and the nitrogen and total phosphorus contents were determined as described^28^.

Water hardness (Ca^2+^ and Mg^+2^) was determined by inductively coupled plasma atomic emission spectrometry (ICP-AES, Varian^®^, model Liberty RL) according to APHA^28^. Fluoride was estimated potentiometrically with an ion-selective electrode, while sulfate was estimated by UV-Vis spectrophotometry (Varian^®^, model Cary 50) according to ASTM^29^. The water samples collected for the metal determinations were preserved by adding nitric acid at pH < 2 (Merck®, Ultrapure acid) and storing them at 4 °C. The samples taken for the determination of the total metal concentrations (manganese and zinc) required acid digestion prior to analysis, while the determination of dissolved metals (total iron and aluminum) required water pre filtering prior to analysis, according to the US Environmental Protection Agency^30^, method 6010C, and measured by atomic emission spectrometry (Varian®, model Libert RL). The uranium concentrations were measured using inductively coupled plasma mass spectrometry (ICP-MS, Perkin Elmer, model NexIOn 300) following APHA methods nos 3030E and 3125B^28^.

All chemical analyses were carried out in the ISO/IEC 17025 environment, since LAPOC is a laboratory accredited by the national accreditation body. The chemical and physical data obtained for the water sampled from the Antas reservoir and for the treated effluent were compared with the limits adopted by the Brazilian guidelines: Conama Resolution 357 Class II^24^, Conama Resolution 430^25^ and Ofício Cnen SLC/50^26^.

### Ecotoxicological Analyses and Toxicity Tests

The *C*. *silvestrii* cultures were maintained at a controlled temperature (24 ± 2 °C) and photoperiod (16:8 h light/dark) in reconstituted water with pH values between 7.0–7.6, electrical conductivity of 160 µS cm^−1^ and hardness between 40 and 48 mg L^−1^ CaCO_3_^31^. The *D*. *magna* cultures were maintained in M4 culture medium with hardness between 175 and 225 mg L^−1^ CaCO_3_, pH 7.0 to 8.0, electrical conductivity of 160 μS cm^−1^, temperature of (20 ± 2 °C) and photoperiod of (16:8 h light/dark)^32,33^. Stock cultures of the green alga *Raphidocelis subcapitata* were maintained in CHU-12 culture medium^34^. The algae were centrifuged and resuspended in an appropriate medium for *C*. *silvestrii* and *D*. *magna*. Both cultured aquatic animals were fed daily on *R*. *subcapitata* chlorophycean algae. For *C*. *silvestrii*, the concentration of the algal suspension ranged from 1 to 5 × 10^5^ cells mL^−1^ per organism, while for *D*. *magna*, the concentration provided at each renewal was of 1 × 10^6^ cells mL^−1^ per adult organism. In addition, the fish food supplement Tetramin®, diluted in processed water (Millipore®, model MilliQ gradient) to a concentration of 5 × 10^3^ mg L^−1^, was offered in 40 mL aliquots. Each aliquot was added weekly to a suspension of dry Fleishmann® yeast at a concentration of 0.2 g per 40 mL of processed water, and stored at 4 °C.

According to international and national standards, the 48 h acute toxicity tests with the reference substances K_2_Cr_2_O_7_ for *Daphnia magna* ^33^ and NaCl for *Ceriodaphnia silvestrii* ^31^, were applied in order to assure that the test conditions were reliable. According to the results of the sensitivity tests carried out, the substance NaCl presented an effective inhibitory concentration (48 h EC_50_) for *C*. *silvestrii* at average values of 1.33 g L^−1^. With respect to *Daphnia magna*, average values of 1.08 mg L^−1^ of the substance K_2_Cr_2_O_7_ provided an effective inhibitory concentration (48 h EC_50_). These values for *C*. *silvestrii* and *D*. *magna* were within the prescribed ranges set by the guidelines.

The 48 h acute toxicity tests using *C*. *silvestrii* ^31^ and *D*. *magna* ^33^ as the test organisms were applied to each water sample from the Antas reservoir (CAB, P41-E and P14) and to the samples of treated effluent (P41-S) before being discharged into the Antas reservoir. In the tests, animals that were less than 24 hours old were divided into four groups (replicates) of five animals each, and exposed to 10 mL (*C*. *silvestrii*) and 50 mL (*D*. *magna*) of each sample in polycarbonate beakers (Brand®). For all the bioassays, control treatments were prepared using dilution water. The experimental conditions (temperature and photoperiod) and dilution waters used in these experiments were the same as those used to culture the organisms. The pH values (Micronal®, model B374 potentiometer), electrical conductivity (Orion®, model 145A conductivimeter) and dissolved oxygen (WTW®, model OXI 316i environmental oximeter) of the effluents and water samples tested were determined at the beginning and end of all the acute toxicity tests.

In addition to the laboratory experiments, 48 h acute toxicity tests with synthetic media, exposing the daphnids (*C*. *silvestrii* and *D*. *magna*) to the following hardness values: 44, 152, 276, 350, 709 and 872 mg L^−1^ as CaCO_3_, were also carried out. The aim of these experiments was to verify the survival potential of these bio-indicators in the high hardness values registered in the environmental samples of the present study. The laboratory tests were carried out with both daphnids in the modified dilution water according to ISO^33^: CaSO_4_.2H_2_O (1500 mg L^−1^); KCl (200 mg L^−1^); NaHCO_3_ (4800 mg L^−1^); MgSO_4_ · 7H_2_O (6100 mg L^−1^), presenting a hardness value of 44 mg L^−1^ CaCO_3_. The higher hardness values (152 to 872 mg L^−1^ as CaCO_3_) were obtained by appropriate dosing of the dilution water with CaSO_4_.2H_2_O.

### Statistical Analyses

The results of the physical and chemical analyses were analyzed using ANOVA and Tukey’s test (post hoc test) to detect significant differences between the water samples obtained from the different sites at the Antas reservoir at the different collection times. The above statistical tests were carried out using the BioEstat 4.0 program^35^. Fisher’s exact test was used to distinguish significant differences in the survival of the cladocerans between the control treatment and the Antas reservoir and treated effluent water samples. The multivariate analysis was used to extract the main characteristics from this large data set. The data were centralized and normalized in such a way that the variable coordinates corresponded to Pearson correlation values with the axes (PC1 and PC2). The PCA analysis was then applied to extract and identify the main sources of variation and the relationship of the variables and samples to these, and hence reduce the amount of data, without losing information^36^.

## Results

### Physical and Chemical Analyses

Table 1 shows the variation in the physical and chemical parameters of the water samples taken from the treated uranium mine effluent and from the Antas reservoir. The average concentrations of the ions determined in the water samples from the treated effluent (P41-S) were as follows: aluminum (0.58 mg L^−1^ ± 0.22), fluoride (15.21 mg L^−1^ ± 9.96), total iron (0.62 mg L^−1^ ± 0.68), manganese (0.80 mg L^−1^ ± 0.52), sulfate (428.51 mg L^−1^ ± 268.59), uranium (0.04 mg L^−1^ ± 0.03) and zinc (0.72 mg L^−1^ ± 1.19). With respect to hardness, the concentrations varied from 543.84 to 1115.15 mg L^−1^. The average values for electrical conductivity and pH were 1137.76 μS cm^−1^ ± 211.05 and 8.20 ± 0.87, respectively. The values for manganese in November 2015 (1.12 mg L^−1^) and in July 2015 (1.38 mg L^−1^); fluoride in February 2015 (12.55 mg L^−1^) and July 2015 (32.06 mg L^−1^) and for pH in October 2015 (9.49) in the samples from P41-S exceeded the limits defined by the Conama Resolution 430^25^.

**Table 1:** Minimum and maximum values found for the physical and chemical variables in the water samples obtained from the sampling sites.

Variables	P41-S	CONAMA/430 Effluent discharge	CAB	P41-E	P14	CONAMA/357 Class II
T (°C)	17.6–25.3		17.9–25.8	17.7–25.0	18.4–25.2	
pH	7.12–9.49	between 5–9	5.79–8.02	6.19–8.56	6.08–7.17	between 6–9
OD (mg L^−1^)	2.9–7.01		3.38–6.11	5.75–7.01	3.29–7.09	>5.0
EC (μS cm^−1^)	981–1500		26–673	66–958	108–547	
Eh (mV)	98–253		201–326	160–362	160–344	
Chla (μg L^−1^)	0.08–1.37		0.28–2.17	0.39–4.12	1.54–3.10	30
Ntot (μg L^−1^)	460.1–714.1		568.4–733.3	309.1–547.3	378.1–761.6	
Ptot (μg L^−1^)	0.75–3.15		1.6–16.45	1.33–15.45	0.70–12.45	50
SS (g L^−1^)	3.6–15.8		1.2–17.3	2.2–6.8	1.7–11.5	
Hardness (mg L^−1^)	543.8–1115.1		6.61–424.2	32.5–883.1	41.49–323.4	
Ca^2+^ (mg L^−1^)	216.9–445.2		1.89–168.0	12.28–351.8	15.89–128.1	
Mg^2+^ (mg L^−1^)	0.342–0.777		0.384–0.949	0.385–0.95	0.39–0.713	
F^−^ (mg L^−1^)	6.50–32.06	10.0	<0.50–1.43	2.21–4.50	1.06–2.37	1.4
${{{\rm{SO}}}_{4}}^{2-}$ (mg L^−1^)	10.9–754.1		3–321.9	12.7–662.4	12.4–253.4	250
Al_d_ (mg L^−1^)	0.37–2.1		<0.1–0.293	<0.10–1.55	<0.1–0.131	
Fe_d_ (mg L^−1^)	0.049–1.58		0.247–1.508	0.156–1.37	0.12–0.31	
Mn (mg L^−1^)	0.006–1.125	1.0	0.302–1.125	0.31–1.0375	0.303–1.21	0.10
U (mg L^−1^)	0.005–0.089		<0.0025–0.005	<0.0025–0.082	<0.0025–0.0145	0.02
Zn (mg L^−1^)	0.017–2.79	5.0	<0.01–0.093	0.02–0.049	0.017–0.034	0.18

Temperature (T), hydrogen potential (pH), dissolved oxygen (DO), electrical conductivity (EC), reduction oxidation potential (Eh), chlorophyll a (Chl), total nitrogen (Ntot), total phosphorus (Ptot), hardness, fluoride (F^−^), sulfate (${{{\rm{SO}}}_{4}}^{2-}$), suspended solids (SS), aluminum (Al), total iron (Fe), calcium (Ca^2+^), magnesium (Mg^2+^), manganese (Mn), uranium (U) and zinc (Zn).

The results obtained using ANOVA for the water samples from the Antas reservoir revealed significant seasonal differences for some chemical and physical variables. The average water temperature during the rainy period (25 °C in November 2014) was significantly higher than that during the dry period (18 °C in July 2015) (*P* < 0.05, Tukey’s test). The average suspended solids contents were significantly higher (*P* < 0.05, Tukey’s test) in October 2015 (11.87 mg L^−1^) than the values observed in July 2015 (2.60 mg L^−1^). The average pH value was significantly higher (*P* < 0.05, Tukey’s test) in July 2015 (7.92) than in November 2014 (6.95), February (6.49) and October 2015 (6.26). The electrical conductivity of the water showed a significantly higher average value in November 2014 (726 μS cm^−1^) than the values observed in February 2015 (67 μS cm^−1^) (*P* < 0.05, Tukey’s test). The highest average value for the reduction-oxidation potential was recorded for water samples in November 2014 (+329 mV), whereas the lowest value was recorded for water samples in July 2015 (+177 mV) (*P* < 0.05, Tukey’s test). With respect to the total phosphorus concentrations, the highest average value was found in the samples collected in February 2015 (14.78 µg L^−1^), whilst the lowest value was found in October 2015 (1.21 µg L^−1^) (*P* < 0.05, Tukey’s test). The average sulfate concentration was significantly higher (*P* < 0.05, Tukey’s test) in November 2014 (412.6 mg L^−1^) than in February 2015 (17.3 mg L^−1^). The lowest average hardness value was found in February (27 mg L^−1^) and the highest in November 2014 (543 mg L^−1^) (*P* < 0.05, Tukey’s test). The average value for manganese determined in November 2014 (0.92 mg L^−1^) was significantly higher (*P* < 0.05) than the values quantified in February (0.38 mg L^−1^), July (0.32 mg L^−1^) and October 2015 (0.43 mg L^−1^). Spatially, the highest fluoride level (2.91 mg L^−1^) occurred in P41-E and the lowest (0.96 mg L^−1^) in CAB and the statistical analysis showed that these two values differed significantly (*P* < 0.05, Tukey’s test).

According to the Conama Resolutions 357 Class II^24^, all the manganese concentrations determined in the water from the Antas reservoir were above the established limit (0.1 mg L^−1^), while in November 2014, the sulfate concentrations at all the sampling stations were above the limit. Uranium exceeded the permitted limit in the water samples from P41-E in November 2014. The fluoride levels exceeded the limits in the samples from CAB in November 2014, from P41-E in November 2014, February, July and October 2015 and from P14 in November 2014 and in February and July 2015^24^. In addition, uranium exceeded the permitted limit (0.008 mg L^−1^) in water samples from P14 in November 2014 and July 2015^26^.

**Figure 2:**
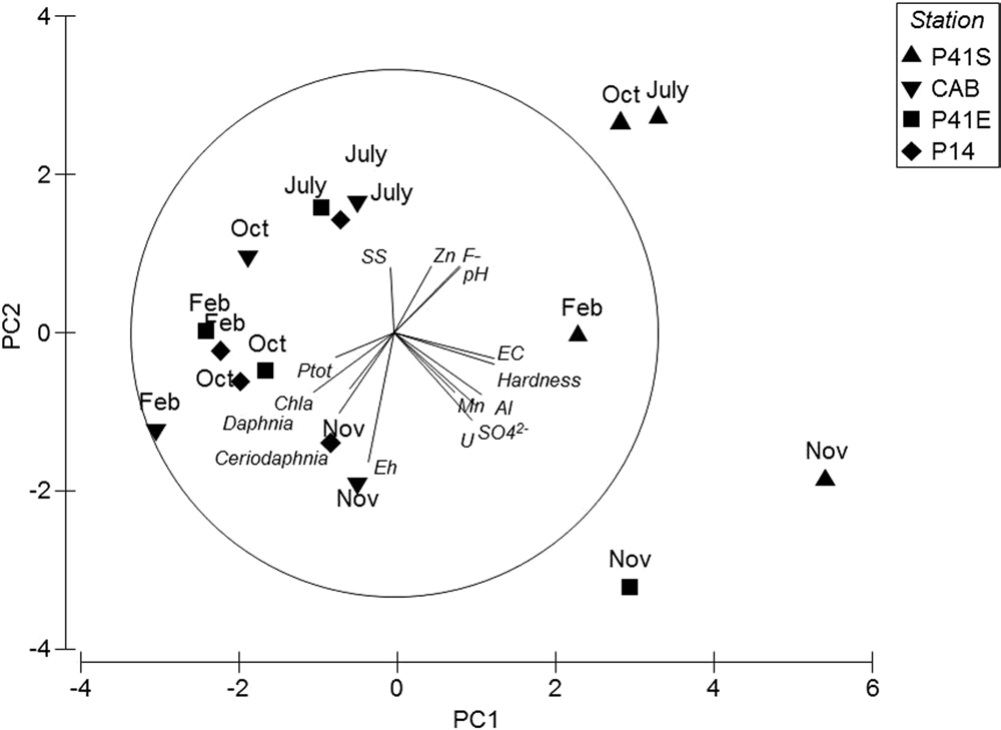
Principal components analysis (PCA) ordination diagram of the sampling sites: P41-S (UTM/INB), CAB, P41-E, and P14 (Antas reservoir) in November 2014 (Nov), February (Feb), July (July) and October (Oct) 2015 for the variables Ptot = total phosphorus, SS = total suspended solids, EC = electrical conductivity, Hardness, ${{{\rm{SO}}}_{4}}^{2-}$ = sulfate, U = uranium, Mn = manganese, Al = aluminum, Eh = reduction oxidation potential, F^−^ = fluoride, Zn = zinc, pH = hydrogen potential and species *Ceriodaphnia silvestrii* and *Daphnia magna*.

The PCA analyses showed that the two axes explained 61.8% of the total variability −42.2% by the first one and 19.6% by the second (Fig. 2). From the PCA it was possible to verify a separation of the samples from the P41-S site in relation to the sampling sites in the Antas reservoir for the physical and chemical variables, throughout the whole period of the study. The samples from the P41-S site indicated the strongest positive correlation with uranium, manganese, aluminum, electrical conductivity, hardness, and sulfate in November 2014. In addition, the same site indicated a positive correlation with pH, zinc and fluoride in July and October 2015. On the other hand, for the sites at the Antas reservoir (CAB, P41-E and P14) it was shown that, in November 2014, these sites indicated correlations with hardness, uranium, electrical conductivity, manganese, aluminum and sulfate values. In addition, in November 2014, the P41-E site showed the strongest positive correlation for these same parameters. In February and October 2015, samples from all the sampling stations were strongly correlated with total phosphorus and chlorophyll *a*.

### Toxicity Tests

**Figure 3:**
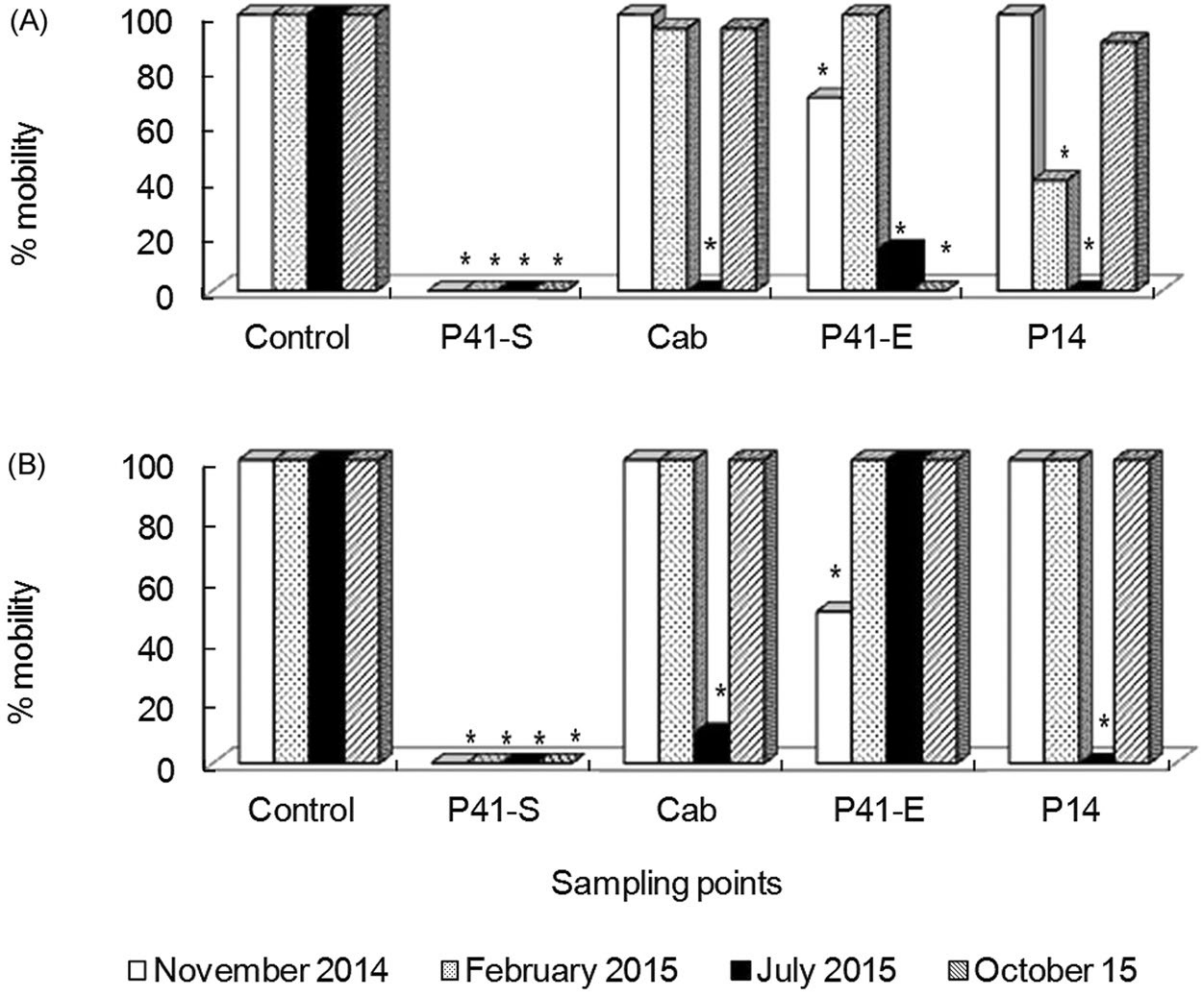
(**A**,**B**) Results from the *Ceriodaphnia silvetrii* (**A**) and *Daphnia magna* (**B**) 48 h acute toxicity tests when exposed to water samples from sampling sites P41-S (UTM/INB) and P41-E, CAB and P14 (Antas reservoir).

Figure 3(A,B) shows the results obtained in the 48 h acute toxicity tests with the treated uranium mine effluent and water from the Antas reservoir. In the toxicity tests, the survival of the control treatments was 100% for *C*. *silvestrii* and *D*. *magna* after 48 h. A statistical analysis of the results from the assays with *C*. *silvestrii* revealed acute toxic effects on the organisms exposed to water samples from the P41-S (all periods), CAB (July 2015), P41-E site (November 2014, July and October 2015) and P14 (February and July 2015) (Fig. 3A). *Daphnia magna* showed acute toxicity when exposed to water samples from the P41-S (all periods), CAB (July 2015), P41-E (November 2014) and P14 (July 2015) (Fig. 3B).

The toxicity assay with *C*. *silvestrii* showed an increase in toxicity from CAB to P41-E in November 2014 and October 2015. On the other hand, a reduction in acute toxicity to *C*. *silvestrii* was detected in the water sample from P41-E in relation to that from P14 site in November 2014 and October 2015. Of a total of 12 samples taken from the Antas reservoir, the frequency of acutely toxic samples was greater for *C*. *silvestrii* (50%) than for *D*. *magna* (25%) and all the samples that showing acute toxicity for *D*. *magna* were also toxic for *C*. *silvestrii*.

According to the PCA (Fig. 2), it was shown that the elevated percentage of mobility of the species *C*. *silvestrii* and *D*. *magna* presented a greater correlation with the water samples from CAB and P14 sites in the month of November 2014 and with the variables of chlorophyll *a* and Eh. In July 2015, considering the sampling sites from the Antas reservoir, the PCA indicated a tendency for a strong correlation with the high percentage of immobility for the species *C*. *silvestrii* and *D*. *magna*. For samples from the CAB in February 2015, P41-E in October 2015 and P14 in February and October 2015, a high correlation was observed with the elevated percentage of mobility of the species *C*. *silvestrii* and *D*. *magna* (100% mobility).

The results of the experiments that evaluated the survival potential of the species *C*. *silvestrii* and *D*. *magna* in the high hardness values registered in the environmental samples (44 to 872 mg L^−1^ CaCO_3_) of the present study, showed 100% of survival in the acute toxicity tests for both daphnids.

## Discussion

This study provided a comprehensive ecotoxicological and chemical evaluation of a tropical reservoir influenced by uranium mining effluents. In the present research, the average values for sulfate, hardness and manganese were higher in November 2014 than in February 2015. The low values for ions in the water from the Antas reservoir in the wet season (February 2015) can be associated with the high rainfall and low water residence time. Rodgher *et al*.^9^ also verified that the Antas reservoir was subject to seasonal variation in relation to the level of chemical compounds. Similar to the present study, Da Silva^37^ found high concentrations of metals and sulfate in a stream close to a mining area in Portugal during periods of reduced rainfall. Gemici^38^ also found seasonal fluctuations in the physical and chemical water quality data for streams in a mercury mine area in Turkey.

With respect to the Antas reservoir drainage area (51 Km^2^) with average flow rates of (2.62 m^3^ s^−1^) and (0.84 m^3^ s^−1^) in the dry and rainy seasons, respectively, it can be seen that it is mainly occupied by natural type vegetation typical of the region, denominated as countryside (79.6%), followed by agriculture (8.7%) and by woods (7.5%). Potentially toxic contaminants arising from agriculture, such as agricultural inputs and pesticides were not detected in this aquatic body. With respect to the presence of potentially polluting industries, only UTM/INB is located in this drainage area^39,40^.

A seasonal environmental diagnosis was carried out by the Authorization Officer and by the Operator (UTM/INB) with respect to the chemical quality of the water in the drainage area of the Antas reservoir, in order to verify the impact of the discharge of the effluent (0.08 m^3^ s^−1^) coming from the treatment plant of this installation, on the Antas reservoir. The results of this study registered significant amounts of the ions fluoride, sulfate, manganese, zinc and uranium in the water. The authors concluded there was evidence of environmental liabilities in the Antas reservoir, caused by operation of the treatment system of the acid waters coming from the UTM/INB^39^.

In the current study, the average suspended solids contents in the Antas reservoir were significantly higher in the wet season (October 2015) than the values observed in the dry season (July 2015). Research has shown that the contribution of suspended matter coming from the banks of the Antas reservoir and the occurrence of resuspension of the material found in the sediment to the water column was much greater in the rainy season than in the dry season, indicating a positive correlation between the rainfall and the suspended matter in the reservoir^9^.

The release of effluents, naturally enriched with metals and radionuclides, is the main legacy of uranium mines. The uranium concentrations found in the Antas reservoir are equivalent to the concentrations measured in aquatic ecosystems impacted by U mining, while the Al, Fe and Mn levels were higher^18^. The results of the physical and chemical parameters determined in the water samples from the Antas reservoir also showed that the sulfate, manganese, fluoride, uranium and aluminum concentrations exceeded the maximum levels according to the Brazilian limits^24,26^. Sediments can also register the historical contamination at the location, as observed by the maximum values recorded in the sediment samples at the Antas reservoir, taken from near the uranium mining effluent discharge zone: F^−^ (0.7 g kg^−1^) Ca^2+^ (22 g kg^−1^) Mn (15 g kg^−1^), U (4 g kg^−1^) and Zn (6.5 g kg^−1^)^39^. The elevated concentrations of the chemical species recorded in the sediment samples at the Antas reservoir revealed contamination by uranium mining effluents, as also observed in the water samples in the current study.

Seasonally, according to the present study, in November 2014 and July 2015 the water samples from the Antas reservoir showed high chemical concentrations (F^−^, U, and Mn), above the limits established by the Conama Resolution 357, Class II^24^ and Ofício Cnen SLC/50^26^, and these metal concentrations were also above the values considered toxic for the zooplankton species registered in the literature^3,19,41–45^. In July 2015, a greater frequency of samples toxic for *C*. *silvestrii* (100%) and *D*. *magna* (67%) was registered in the Antas reservoir, when compared to the results obtained in the other sampling periods. To the contrary of that expected, the smallest number of toxic samples was registered for both species in November 2014, only 25%. Based on the principal components analysis (Fig. 2), the values for water hardness probably controlled the results obtained in the toxicity tests in November 2014 and July 2015. In November 2014, the extremely high average value for hardness (543.55 mg L^−1^ CaCO_3_), registered in the water samples from the Antas reservoir, possibly reduced the toxicity potential of this chemical species mixture with respect to the zooplankton bioindicators. On the other hand, in July 2015, when elevated concentrations of the chemical species were registered and the average value for hardness indicated soft water (59.22 mg L^−1^ CaCO_3_), acute toxicity was detected for both *C*. *silvestrii* and *D*. *magna*. In the literature there is little information related to the influence of water hardness in the expression of metal toxicity in environmental samples. The results for acute toxicity registered in the present study in November 2014 and July 2015 confirmed those of Yim *et al*.^46^, who demonstrated that the toxicity of metal mixtures to daphnids was greatly increased in soft water (44 mg L^−1^ as CaCO_3_) in relation to the results obtained in a hard water test solution (150 mg L^−1^ as CaCO_3_). It is known that water hardness affects the toxicity of various metals in many aquatic species^47,48^.

Water hardness is a chemical characteristic that depends on the occurrence of alkaline earth metals, mainly calcium and magnesium elements. This feature is due to the dissolution of minerals in soils, and can also be associated with pollution by industrial effluents^49^. It has also been suggested that hard waters, where there are greater Ca^2+^ ion concentrations than Mg^2+^ ion concentrations, offer greater protection to aquatic life with respect to metal toxicity, than aquatic bodies where the greater part of the hardness is due to Mg^2+^ ions^50^. In the present study the hardness water values were related to the treatment of the *in natura* effluent coming from UTM/INB with slaked lime to neutralize the DAM, producing CaSO_4_. Thus the greater part of the hardness in the Antas reservoir was due to Ca^2+^, indicating that this element could have acted as a protective factor for the test species in the water samples in November 2014, and could thus have influenced the expression of toxicity. The survival of *C*. *silvestrii* and *D*. *magna* was observed in samples from the Antas reservoir with extremely high hardness values (323.4 to 424.2 CaCO_3_ mg L^−1^ at sites P14 and CAB, respectively) in November 2014, which has still not been registered in the literature. In addition, the high hardness results found in the laboratory experiments carried out in the current study (152 to 872 mg L^−1^ CaCO_3_) were in accordance with those registered in environmental samples, showing 100% survival for both cladocerans. Thus the results indicated that extremely high hardness values were not limiting for the survival of the cladocerans. Acute toxicity for *D*. *magna* has already been reported after exposure to hard water containing 600 mg L^−1^ of CaCO_3_^51^.

Major cations may compete with trace metals such as fluoride and sulfate for target cell sites and are known to modify their toxicity for aquatic organisms^44,52^. Kinraide *et al*.^53^ demonstrated that increasing concentrations of cations (e.g. Ca^2+^, Mg^2+^) in the exposure medium decreased the negative electrical potential at the cell membrane and hence reduced the electrostatic attraction of the cell membranes for metal ions and decreased the toxicity. On the other hand, Goulet *et al*.^19^ suggested that Ca channels in the cell membranes did not compete with uranium metal for the same uptake sites. Instead it appeared that the increase in Ca, Mg and sulfate increased the tolerance of the aquatic organisms to U. Davies and Hall^54^ determined that ${{{\rm{SO}}}_{4}}^{2-}$ toxicity was inversely related to increasing Ca^2+^ concentrations and Ca:Mg ratios in tests with *D*. *magna*. Similarly, Shamsollahi *et al*.^55^ found that fluoride ions were less toxic to *D*. *magna* in hard waters.

Data from the present study clearly showed that the variation in water hardness considerably altered the toxicity of the Antas reservoir samples to the cladocerans during the year. The toxicity bioassay with *C*. *silvestrii* indicated an increase in acute toxicity from CAB to P41-E. On the other hand, a reduction in acute toxicity for *C*. *silvestrii* was detected in water samples from P41-E in relation to water from the P14 site. These acute toxicity results agreed with the spatial distribution of the concentrations of uranium, sulfate and fluoride, and with the electrical conductivity values at P41-E (near the site where the mining effluent was discharged), which were higher than those at CAB and P14. In this study the toxicity bioassays indicated that a large proportion of the water samples collected at P41-E and P14 had toxic effects on *C*. *silvestrii*, possibly resulting from the input of uranium mine treated effluent (P41-S) into the Antas reservoir. The treated effluent samples were positively correlated with elevated concentrations of uranium, manganese, aluminum, zinc and fluoride and with high electrical conductivity and pH values. In addition, the results of the acute toxicity registered at P41-S did not conform with the standards for the discharge of effluent into the environment, according to the Conama Resolution 430^25^. The current Brazilian Conama Resolution 430^25^ prescribes that the effluent of a polluting source must not cause or present potential to cause toxic effects to aquatic organisms in freshwater bodies. The concentrations of the chemical species (manganese, fluoride, sulfate and uranium) at the following sites: P41-S (treated effluent before being discharged into the environment), P41-E and P14 (Antas reservoir), may represent a risk to invertebrate species.

Thus one must discussion toxicity for aquatic invertebrates under different physical and chemical conditions, since environmental variables (e.g. water hardness) have an influence on metal and ion toxicity in freshwater systems^56^. The environmental variables (e.g. hardness, alkalinity, pH, chloride) which contribute substantially to modifying the toxicity, can be related to the bioindicator species applied in the toxicity tests (e.g. *Hyalella azteca*, *Ceriodaphnia silvestrii Lemna minor*, *Chironomus dilutes*, *Clorella* sp.), the different uptake pathways and the physical and chemical composition of the water^19,42,44,45,48,57–59^.

Freshwater invertebrates in general and daphnids in particular, have been widely used in ecotoxicological studies of freshwater reservoirs contaminated with anthropogenic toxic substances from mining activities. Mejía-Saavedra *et al*.^43^ showed that the river that receives the waters coming from the mining area in Salado, Mexico, was acutely toxic to cladocerans. Chen *et al*.^17^ also demonstrated that mining effluent made the water of the Pearl River (China) highly toxic to daphnids. Water hardness is a very variable factor, depending on which geochemical region of the world is involved. *Daphnia magna* is a native microcrustacean from Europe, where the natural water contains high carbonate values^60^. Thus *D*. *magna* was ecologically relevant to this study area because of the high hardness values recorded in the uranium mine effluent and in the water of the Antas reservoir. However, the geographical distribution of *D*. *magna* is primarily limited to areas of high and middle latitudes^61^. From the viewpoint of ecotoxicity bioassays, it is more adequate to use native species than to import non-indigenous species. In Brazil, the cladoceran *C*. *silvestrii* is a test species recommended for ecotoxicological evaluations^31^ and in the present study *C*. *silvestrii* was more sensitive than *D*. *magna* to acute exposures to the environmental water samples. These results confirm the importance of carrying out acute toxicity tests using an indigenous species such as *C*. *silvestrii* in the ecotoxicological assessment. The survival potential of *C*. *silvestrii* in a tropical aquatic environment around a mining area with very high hardness values, has still not been registered in the literature, but was confirmed by the results of the laboratory tests carried out in this study, indicating the suitability of this tropical cladoceran species as a test organism.

This study highlights the usefulness of the ecotoxicological approach associated with the chemical approach as an effective tool to evaluate critical areas within aquatic ecosystems potentially impacted by U discharges into the environment. A clearly protective effect to the acute toxicity of the metals in the water from the Antas reservoir was provided to the cladocerans *C*. *silvestrii* and *D*. *magna* by the very high hardness values. *C*. *silvestrii* is a native and widely distributed cladoceran throughout South America, and was shown to be sensitive to detecting toxic conditions in water samples from the Antas reservoir.
